# An atypical presentation of Wernicke-Korsakoff encephalopathy mimicking a left hemispheric stroke: case report

**DOI:** 10.3389/fpsyt.2024.1499803

**Published:** 2024-12-20

**Authors:** Ioana Butnariu, Daniela Nicoleta Anghel, Adela Magdalena Ciobanu, Florentina Cojocaru, Dana Antonescu-Ghelmez, Maria Mirabela Manea, Florian Antonescu

**Affiliations:** ^1^ Department of Neurology, “Carol Davila” University of Medicine and Pharmacy, Bucharest, Romania; ^2^ Department of Neurology, National Institute of Neurology and Neurovascular Diseases, “Carol Davila” University of Medicine and Pharmacy, Bucharest, Romania; ^3^ Department of Psychiatry, Prof. Dr. Alexandru Obregia Clinical Psychiatry Hospital, Bucharest, Romania

**Keywords:** Wernicke-Korsakoff, encephalopathy, thiamine, stroke mimic, case report

## Abstract

**Background:**

Wernicke-Korsakoff encephalopathy is a metabolic disease caused by vitamin B1 deficiency that predominantly affects alcoholic patients. Its clinical picture is characterized mainly by altered mental status with memory deficits, ophthalmoparesis, and ataxia, although other clinical manifestations may also be present. The current case presents certain clinical difficulties regarding the diagnosis when confronting an atypical presentation of a classical disease in an acute setting when a decision to administer an intravenous thrombolytic agent needs to be made.

**Case presentation:**

This case involves a young male patient, with a history of chronic alcohol abuse, malnourished, and in poor general health, who presented with right-sided hemiparesis and language disturbance of acute onset, suggesting a left hemispheric stroke. The psychological examination was difficult due to a mix of confusion and aphasia, the latter being challenging to assess as the patient had a dismissive and highly deflective attitude toward the examiner. The initial cerebral computed tomography scan was unremarkable, in line with early imaging in ischemic stroke patients. On subsequent magnetic resonance imaging, lesions were observed in the medullo-pontine tegmentum, around the aqueduct of Sylvius, in the mamillary bodies, in the medial thalami, but also extensive bilateral cortical involvement in the frontal lobes. After receiving intravenous vitamin B1 treatment, the patient made a slow, but full, recovery, after eight weeks of hospitalization, and was subsequently transferred to a psychiatry clinic for treatment of his addiction.

**Conclusion:**

Atypical Wernicke-Korsakoff encephalopathy can closely mimic stroke, usually akin to a vertebro-basilar lesion. Our case is the first report we are aware of Wernicke-Korsakoff encephalopathy feigning a left hemispheric stroke with aphasia and right hemiparesis. This has implications for the emergency medicine doctor, neurologist and the stroke specialist when considering an emergency differential diagnosis for a patient with an initial normal computed tomography scan, especially in regard to deciding acute therapy.

## Background

Wernicke-Korsakoff encephalopathy (WKE) is a severe, potentially life-threatening disease that affects individuals with thiamine (vitamin B1) deficiency. It usually occurs in chronic alcoholics due to a combination of low intake and increased consumption. Nevertheless, it has also been reported in other situations such as gastrointestinal surgery, hyperemesis gravidarum, liver disease, hyperthyroidism, prolonged voluntary fasting, and severe anorexia nervosa (1–4). It is frequently underdiagnosed, mainly due to the significant heterogenicity of its presentation (5). The classical clinical triad consists of altered mental status with severe memory impairment, ataxia, and ophthalmoparesis. Nevertheless, the simultaneous occurrence of all the symptoms at the initial presentation is not universal, the typical clinical scenario being found in only 16 to 33% of patients (1).

Typically, the changes in the mental status of patients consist of confusion and amnesic dysfunction, especially concerning the formation of new memories. Drowsiness, apathy, agitation, behavioral disturbances, and hallucinations can be present (1, 6). Oculomotor abnormalities are represented by nystagmus, usually horizontal, and bilateral weakness of abduction, which can progress to complete ophthalmoplegia (6–8). Other rare clinical findings reported in the literature are bilateral acute vision loss, severe global amnesia, unilateral facial palsy, hemiparesis, hypothermia/hyperthermia, deafness, seizures, and high-output congestive heart failure (1, 9–13).

The acute onset of symptoms can mimic a stroke, usually in the vertebrobasilar artery territory, especially since nonenhanced computed tomography (CT) imaging has a low sensitivity for visualizing hyperacute and early acute ischemic lesions (14).

The diagnosis is supported by magnetic resonance imaging (MRI) and a significant response to treatment with high doses of thiamine. Rapid treatment is of paramount importance because it is the only way to limit permanent neurological damage and subsequent disability (7). If the neurological deficits are left untreated, they become irreversible, and the mental status continues to decline, leading to dementia and even death (7).

## Case presentation

We present the case of a right-handed 36-year-old male, a heavy smoker, living alone, with poor dietary habits and a long history of daily alcohol consumption who was admitted to our clinic for right-sided hemiparesis and aphasia of acute onset. The patient was found in the above-mentioned state in the morning, having last been seen in an apparently normal state of health during the previous evening.

On admission, the patient was lethargic and disoriented, with a right-side hemiparesis 3/5 on the Medical Research Council (MRC) scale more pronounced in the upper limb, moderate mixed aphasia, partial conjugate gaze palsy toward the right side without forced gaze deviation, slight horizontal nystagmus, and singultus. Head impulse test could not be performed adequately due to lack of cooperation. His deep tendon reflexes were diminished, and no pathological reflexes were obtained. Sitting was unimpaired, walking was limited by the right hemiparesis, but could be achieved with unilateral support. No limb or truncal ataxia were observed. His speech was disorganized, with decreased fluency and object-naming difficulties. His understanding was significantly impaired, but he was able to perform simple commands occasionally. Nevertheless, the fragmented contact that was established was also marred by cognitive and behavioral problems. The patient was avoidant, deflecting the examiner’s questions and occasionally inserting delusional statements. Structured cognitive testing was not possible at this point. Retention of new information seemed to be low. Confabulations were not observed.

The results of routine blood tests were unremarkable except for mild leukocytosis and thrombocytopenia. The blood level of cyanocobalamin was normal, as was his cerebrospinal fluid exam. Tests for human immunodeficiency virus, syphilis, hepatitis B and C were negative. Unfortunately, serum thiamine levels and erythrocyte thiamine transketolase activity were not readily available in the emergency department of our hospital, nor were they requested initially, as, due to the acutely developed focal neurological deficits, the patient was at that time suspected of having a stroke. Later, as the diagnosis became clear and his evolution was already favorable under vitamin supplementation, they were no longer significant.

**Figure 1:**
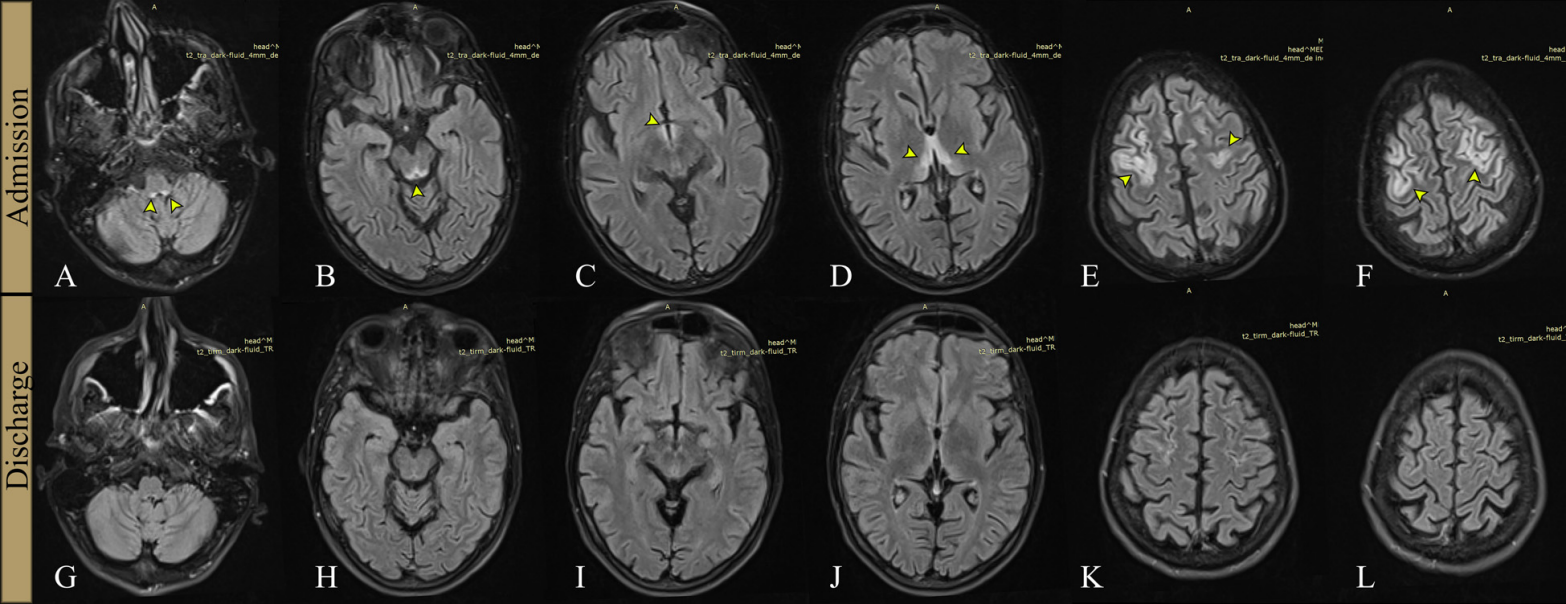
Cerebral MRI examinations at admission and discharge. FLAIR sections at admission **(A–F)**, respectively at discharge, 8 weeks later **(G–L)**. Hyperintense signal (arrows) is visible on the first examination, in the medullo-pontine tegmentum **(A)**, around the aqueduct of Sylvius **(B)**, in the mamillary bodies **(C)**, medial thalami **(D)**, and in the frontal cortex bilaterally **(E, F)**. The second examination **(G–L)** shows marked improvement of the signal changes in initially affected areas.

**Figure 2:**
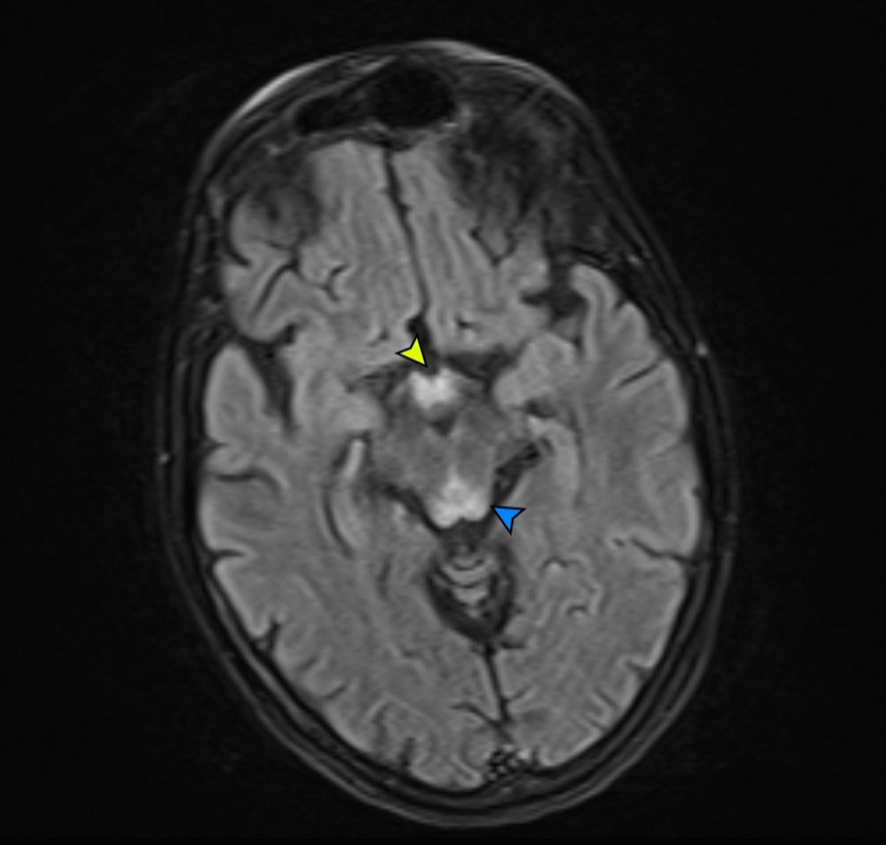
Mamillary bodies involvement. FLAIR axial image at admission, showing increased signal in the mammillary bodies (yellow arrow) and the posterior midbrain (blue arrow).

A brain CT scan on admission showed only cerebral atrophy, but this was interpreted as consistent with an acute ischemic lesion. In the past 18 hours, he had experienced no obvious deficits, and a pure cerebral cortex necrosis would not have been visible on the CT scan. Next, a cerebral MRI scan was ordered, showing hyperintense fluid-attenuated inversion recovery (FLAIR) lesions in the medullo-pontine tegmentum, around the aqueduct, in the mamillary bodies, medial thalami, and significant cortical lesions in both frontal lobes (**Figures 1A–F**, **2**).

**Figure 3:**
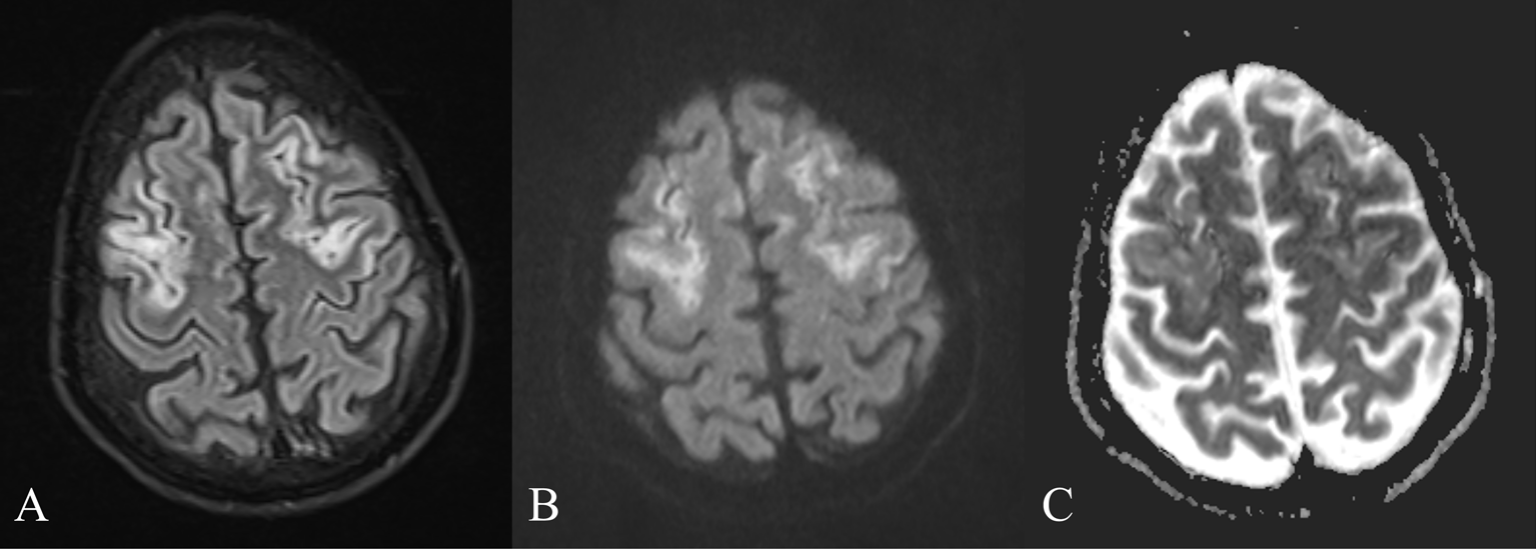
FLAIR, DWI and ADC. **(A)** FLAIR image showing increased signal in the frontal cortex, bilaterally. **(B)** Increased DWI signal is present in the same areas. **(C)** ADC map without a corresponding low signal, ruling out an ischemic stroke.

The lesions had variable degrees of diffusion-weighted imaging (DWI) hyperintensity but without a corresponding low signal on apparent diffusion coefficient (ADC) (**Figure 3**), thus not consistent with an ischemic lesion, probably representing T2 shine through.

The periaqueductal and symmetrical mamillary bodies and thalamic lesions (**Figures 1B–D**, **2**) in the context of his chronic alcoholism were the main reasons for taking into consideration a severe WKE.

Being undernourished, with oculomotor anomalies, including nystagmus and confusion our patient fits the Caine criteria for WKE, though the motor deficit and proeminent language disorder were definitely atypical in our experience (15).

**Figure 4:**
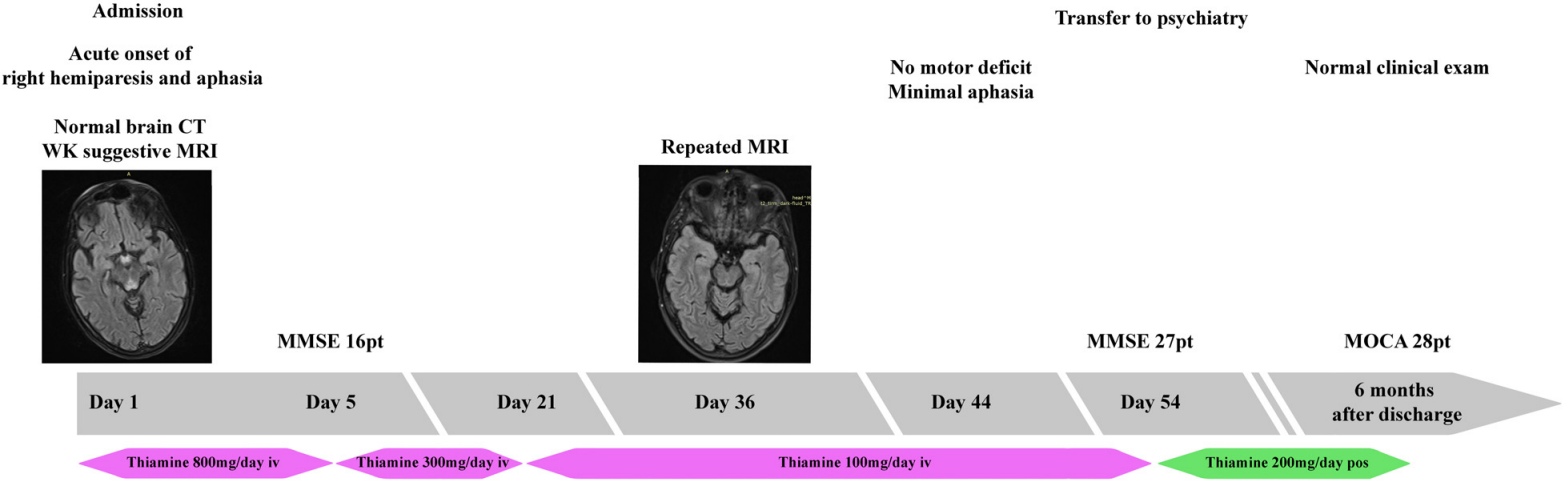
Timeline of the clinical episode. The progression of the patient’s episode organized as a timeline.

He was started on treatment with intravenous thiamine, 800mg per day, and other vitamin supplements, under which he gradually improved, with progressive remission of the aphasia in approximately two weeks. As the language improved, memory deficits and unstructured delirium became increasingly evident. The cognitive deficits improved more slowly, but they improved over the next six weeks. As cognitive testing became possible, we initially obtained a Mini Mental Status Examination (MMSE) (**Figure 4**) score of 16 which would improve to 27 points at discharge, eight weeks after admission.

The motor deficit improved gradually with complete remission in approximately six weeks and there were no adverse events during treatment. An MRI examination repeated one month after admission revealed significant regression of the lesions, as shown in (**Figures 1G–L**). He was then transferred to a psychiatry clinic to treat his alcohol addiction and, at the six-month follow-up, he had a normal neurological exam and cognitive testing. He had stopped drinking completely and was adjusting well.

## Discussion and conclusions

The continuous increase in the stroke thrombolysis rate and decrease in door-to-needle time in many countries were also associated with an increased risk of using thrombolytic therapy in stroke mimics (16). Patients with WKE as a stroke mimic have been reported before, but this is the first case we know of simulating an acute left hemispheric lesion with an atypical clinical presentation with right-side hemiparesis and aphasia, conjugate gaze palsy limited toward an apparent left frontal lobe lesion and lack of ataxia (12). Retrospectively, the memory deficits were surely present from the start, but they were difficult to assess due to the language problems. Even though motor deficits have been described in WKE, they usually presented as tetraparesis, since the lesions are generally bilateral (17, 18). In cases where hemiparesis was reported, it was not associated with other signs of hemispheric deficits, presenting more often with diplopia, complex ophthalmoplegia or ataxia, and mimicking a vertebro-basilar stroke. Other reports of aphasia or unilateral hemiparesis are very few, with one case with acute onset of aphasia and limb ataxia in which the diagnosis was not supported by specific MRI changes and the second one presented with an acute confusional state, double vision and slight hemiparesis with normal speech and language (19). In the second case, typical WKE lesions in the medial thalamus and contrast enhancement of the mamillary bodies were associated with a small area of restricted diffusion of the left precentral gyrus, but that could have been a postictal marker, as the patient also presented generalized tonic-clonic seizures (20). Another case presented with stroke-like left occipitotemporal cortical lesions similar to those encountered in mitochondrial encephalomyopathy with lactic acidosis and stroke-like episodes (21).

While not typical, auditory impairment has been occasionally described in WKE and can contribute to the overall encephalopathy. The proposed mechanisms vary, from bilateral sensorineural hearing loss to more complex anomalies secondary to lesions in the thalamus or the inferior colliculi with impaired multisensory audio-visual integration of speech and/or impairment of word discrimination, progressing up to word deafness (22–24). Our patient had apparently unaffected hearing at the initial examination. Since cortical involvement was present, in addition to the thalamic and posterior midbrain lesions, it is probable the aphasia was of mixed origin.

It has been proposed that horizontal gaze-holding failure, as revealed by the head impulse test, could be an important diagnostic marker in WKE, especially in cases with an incomplete clinical picture (25).

The correlation between alcohol consumption and the decrease in thiamine availability has long been established. It has been proven that alcohol harms thiamine absorption and storage (26). Additionally, in alcoholic patients, the phosphorylation of thiamine is impaired in both the central and peripheral nervous systems (27). Thiamine is a key cofactor for three enzymes: transketolase, pyruvate dehydrogenase (PDH), and alpha-ketoglutarate dehydrogenase (α-KGDH) which have complex implications in metabolic circuits with roles in adenosine triphosphate (ATP), neurotransmitters (acetylcholine, glutamate, gamma-aminobutyric acid) and myelin synthesis. Decreases in the activity of PDH and α-KGDH can result in reduced ATP synthesis, increased oxidative stress, glutamate-mediated excitotoxicity, apoptosis, and various degrees of inflammation, which in turn can contribute to cell damage and death (23–25). Histologically, cytotoxic and vasogenic edema are present initially, and necrosis ensues if the deficit persists. Impaired energy metabolism induces dysfunction of the cell electrolyte homeostasis and accumulation of lactate, alanine, and glutamate at cytotoxic levels, processes that result in cytotoxic edema. In conjunction with the low pH and glutamate excitotoxicity, ATP depletion also damages the astrocytes, and the brain-blood barrier becomes dysfunctional, with associated vasogenic edema (27–30).

The MRI is of particular importance in the diagnosis of WKE and differential diagnosis. It usually reveals bilateral and T2/FLAIR hyperintensities, with a symmetrical distribution affecting the medial thalami, mamillary bodies, and periaqueductal region. As was the situation in our case, anomalies in these areas are typical and can help in establishing the correct diagnosis even if extensive cerebral involvement is also present (31, 32). Additionally, similar lesions in the brainstem, cerebellum, thalamus, red nuclei, caudate nuclei, splenium, and cerebral cortex have been described in the literature in alcoholic and nonalcoholic patients (11, 33). Atrophy of the mamillary bodies may be absent initially but is a typical finding (34).

DWI/ADC can show variable patterns. Cytotoxic edema in ischemic stroke presents with a high DWI signal, with corresponding low ADC, but up to 6.8% of acute ischemic stroke can be DWI negative (35). Unlike acute ischemic stroke, in WKE, this high DWI signal does not always correspond to irreversible neuronal damage, and part of DWI hyperintensity could be due to the T2 shine-through effect (31, 32). In both stroke and WKE, the mechanism of the cytotoxic edema is impaired cellular energy metabolism, which restricts membrane transport of water and electrolytes. Nevertheless, in WKE, the slow development of metabolic dysfunction allows the cells to adapt and increases survival time. Additionally, the pentose phosphate shunt pathway, which has a neuroprotective role in acute ischemia, seems to remain functional in WKE even when the thiamine deficit is severe, constituting a metabolic reserve for the affected cells (36, 37).

DWI may show only slightly increased signal intensities within the thalami and periaqueductal area, but no significant changes in signal intensity in the other affected brain areas (32). In most cases of acute ischemic stroke in the corresponding areas of high DWI, there are decreased ADC values. In WKE patients the ADC in lesional areas has a variable pattern, from normal to various degrees of reduction, but generally less than that in most cases of ischemic stroke, although there is no clear cutoff value to differentiate them (24, 27).

MRI can also reveal areas with high ADC signal and variable DWI signal, which reflect vasogenic edema secondary to the dysfunction of the blood-brain barrier (BBB) (38). Contrast enhancement can be present in approximately half of the cases, revealing the areas where the BBB is severely disrupted (31). These MRI anomalies tend to regress with adequate treatment (39–42).

In our patient, lesions were also present in the high frontal cortex bilaterally, a distribution that is rarely encountered. Available data suggest that patients with cortical lesions have a worse prognosis, especially when contrast enhancement is present (43). Despite the severe clinical and radiological findings at admission, he made an excellent, even if delayed, recovery with complete remission of motor, language, and cognitive deficits in approximately eight weeks.

Albeit slowly, the patient has made a complete clinical recovery. We have discussed his experience with him in multiple instances. Retrospectively, he describes the initial weeks as “surreal”, with many memory “holes” and a dream like quality of perception. He finds it difficult to believe that this has happened to him and seems sincerely grateful to the medical team for aiding in his recovery. He has stopped drinking, and acknowledges his alcohol addiction, for which he continues to see a psychiatrist. He continues to smoke and affirms a lack of desire to quit. He routinely tends to play down the severity and the potential consequences of the episode. On the positive side, the illness has brought about a reconciliation with his estranged father, who assumed charge of his care after he left the hospital.

Our case is an illustration of the heterogenicity of WKE clinical presentation. There are very few reports of aphasia or unilateral hemiparesis due to WKE, and, to our knowledge, no case has been reported as presenting with both simultaneously, mimicking a left hemispheric stroke (20, 44).

These findings have implications in the management of such patients, given that thrombolysis and other revascularization strategies could be considered. A lack of efficacy of thrombolytic treatment and the absence of an arterial occlusion should trigger a cerebral MRI exam, especially in patients with a history of alcohol abuse or malnutrition even if they present with language disturbances or focal motor deficits (12). Such an atypical presentation can lead to delays in the introduction of B1 vitamin treatment in the required dosage.

Usually, cortical involvement in WKE carries a reserved prognosis; however, as our case shows, if properly and early treated, a complete recovery is possible.

## Data Availability

The original contributions presented in the study are included in the article/supplementary material. Further inquiries can be directed to the corresponding author.
